# Methylphenidate leads to disruptions in rest/wake patterns after discontinuation

**DOI:** 10.1007/s00213-025-06859-y

**Published:** 2025-07-30

**Authors:** Carolyn Cueto, Magdalena R. Gonzales, Alexandra N. Tejada, Kimberly Guerrero Leon, Alexandra Mora, Andrew Cabrera, Leslie R. Amodeo

**Affiliations:** Department of Psychology, California State University San Bernardino, San Bernardino, CA 92407 USA

**Keywords:** Adolescence, Alcohol, Sleep, Activity rhythms, Locomotor

## Abstract

Children with attention-deficit/hyperactivity disorder (ADHD) experience more sleep problems than their peers. As stimulant medications, particularly methylphenidate (MPH), are the most common treatment for pediatric ADHD, there has been growing interest in whether such medications contribute to sleep disturbances and the development of sleep disorders later in life. Despite ongoing interest, evidence related to MPH on sleep functioning in children remains mixed. The present study investigated the effects of MPH on 24-hour rest/wake activity patterns and circadian rhythms in adolescent versus adult rats. Male and female Long-Evans rats were administered MPH (1 or 2 mg/kg, i.p.) or control twice daily for 10 days during either adolescence (PD 30–39) or adulthood (PD 80–89). Non-invasive activity monitors, secured to each rat using custom-fitted jackets, were used to assess circadian rhythms and detailed activity patterns over a 24-hour reverse light/dark cycle. Microanalysis of activity patterns were assessed on the last day of MPH treatment, during acute discontinuation, and after 10 days of prolonged discontinuation. Results showed that repeated MPH administration produced dose- and age-specific increases in activity across the light/dark cycle on the last day of treatment. In adults, this was associated with longer and more frequent active episodes during the dark period, and transient increases in fragmented activity during early withdrawal. MPH also impaired rest quality in female rats, reflected by fewer rest episodes and increased fragmentation during the light period. While some of these disruptions persisted immediately after discontinuation, more pronounced impairments in rest quality emerged after 10 days of withdrawal. These findings suggest that while MPH may enhance activity during treatment in an age- and sex-dependent manner, its discontinuation may lead to lasting reductions in sleep quality in both adolescents and adults who are transiently exposed to the psychostimulants.

## Introduction

Children with attention-deficit/hyperactivity disorder (ADHD), whether taking stimulant medications or not, experience more sleep problems than their peers (Becker et al. 2016). Although clinicians have recognized the relationship between ADHD and sleep disturbances for over 60 years (Laufer and Denhoff 1957), significant gaps remain in our understanding. Among children with ADHD, up to 70% experience sleep-related issues, with even higher prevalence observed in girls with ADHD (Becker et al. 2018). The most common sleep problems include difficulty initiating sleep (Cortese et al. 2009), poor sleep quality or fragmented sleep (Bondopadhyay et al. 2022), and shortened sleep duration compared to typically developing peers (Becker 2020; Bondopadhyay et al. 2022; Darchia et al. 2022). Individuals with ADHD are also at an eightfold higher risk for sleep disorders, with the highest rates occurring in middle-aged and older adults, suggesting that risk may increase across the lifespan (Ahlberg et al. 2023). These nighttime disturbances often lead to excessive daytime sleepiness, which may exacerbate the behavioral symptoms commonly associated with ADHD (Eyuboglu et al. 2017); Craig et al. 2020; Becker 2020). Given the extent of both daytime and nighttime symptoms, it is increasingly clear that ADHD should be conceptualized as a disorder that exhibits symptoms across the entire 24-hour period (Becker 2020).

Because stimulant medication is the most common treatment for pediatric ADHD, there has been long-standing interest in whether these medications exacerbate existing sleep disturbances or contribute to the development of sleep disorders later in life (for reviews, see (Konofal et al. 2010; Stein et al. 2012). Recent increases in ADHD diagnoses and subsequent psychostimulant prescriptions during the COVID-19 pandemic highlight the need to further investigate the effects of stimulant treatment in youth with milder ADHD symptoms (Kazda et al. 2021; Danielson et al. 2023). One of the most prescribed stimulants is methylphenidate (MPH), which has been used worldwide for over 60 years to treat children and adolescents with ADHD. MPH has been shown to enhance attention and response inhibition and reduce hyperactivity in patients with ADHD(Storebø et al. 2015, 2023), as well as in non-clinical populations and animal models (Kuczenski and Segal 2002). However, children with ADHD who are treated with MPH often experience delayed sleep onset, insomnia, and reduced rapid eye movement (REM) sleep (Galland et al. 2010). MPH treatment can also disrupt sleep/wake rhythms, including changes in circadian amplitude, phase shifts, and alterations in locomotor behavior in children with ADHD (Corkum et al. 2008; Ironside et al. 2010). Despite ongoing interest, evidence on how MPH affects sleep remains conflicting (Becker et al. 2016).

Animal studies have further demonstrated the impact of MPH on sleep/wake activity and circadian rhythms. In sleep-deprived adult mice, acute MPH (10–30 mg/kg, p.o.) increases sleep latency and extends wakefulness (Ishida et al. 2010; Antle et al. 2012). In adult male rats, acute MPH (0.1–5.6 mg/kg) causes EEG desynchronization during wakefulness, marked by decreased power in the alpha, beta, and gamma frequency bands (Zanettini et al. 2019). Repeated MPH exposure alters daily locomotor activity rhythms in adult (Gaytan et al. 1997; Algahim et al. 2009, 2010; Lee et al. 2011; Trinh et al. 2013) and adolescent rats (Dafny and Yang 2006; Yang et al. 2006; Lee et al. 2009; Bergheim et al. 2012). Moreover, adult rats appear more sensitive to MPH-induced circadian disruption compared to those exposed during development (Kayyal et al. 2015). Developmental differences are also evident in sensitivity to catecholaminergic agonists, with adult rats showing heightened behavioral responses to psychostimulants relative to juveniles (Spear and Brake 1983). In young adult male mice, MPH treatment has been shown to delay the onset of suprachiasmatic nucleus (SCN) electrical activity (Antle et al. 2012) and alter clock gene expression (Baird et al. 2013), both of which disrupt sleep and circadian rhythms. Even short-term circadian disruption can contribute to long-term health conditions such as diabetes, cardiovascular disease, and hypertension (Karlsson et al. 2001; Bray and Young 2007; Lamont et al. 2007). Thus, changes in circadian rhythmicity may serve as early markers of the potential long-term impact of psychostimulant exposure (Algahim et al. 2010; Bergheim et al. 2012).

Despite substantial human data linking MPH to sleep pathology, the causal relationship between MPH and sleep disruption in a non-diseased model remains unclear. This study examines age- and sex-specific effects of MPH on 24-hour activity rhythms in male and female rats during adolescence and adulthood. To our knowledge, this is the first study to assess patterns of sleep-wake activity both during and after discontinuation of MPH treatment. We have recently validated a non-invasive activity monitoring system (FitBark^®^) for evaluating activity rhythms in rats (Ehlers et al. 2023; Amodeo et al. 2025), which offers a more translatable and convenient method for assessing sleep-wake behavior in naturalistic environments. This system allows adolescent rats to be monitored in their standard colony housing, enhancing ecological validity.

In this study, male and female Long-Evans rats were administered MPH (1 or 2 mg/kg, i.p.) or control twice daily for 10 days during either adolescence (P30–39) or adulthood (P80–89). Activity was recorded on the final day of treatment, 24 h after treatment cessation (acute withdrawal), and 10 days post-discontinuation (prolonged withdrawal). Results indicate that repeated MPH administration leads to dose- and age-dependent increases in activity across both light and dark phases on the final day of treatment. Although some changes in rest quality were observed immediately following treatment cessation, significant rest disruptions emerged in all groups after 10 days of withdrawal. These findings suggest that while MPH increases activity in an age- and sex-specific manner during treatment, prolonged discontinuation may lead to reduced sleep quality in both adolescent and adult animals exposed to psychostimulants.

## Materials and methods

### Subjects

The study was composed of 52 adult and 56 adolescent Long-Evans rats born at California State University, San Bernardino with breeding pairs originating from Charles Rivers. Litters were culled to 10 per litter, with even numbers of males and females when possible. Rats were weaned at postnatal day (PD) 23 and housed with their same-sex littermate in groups of 2–3 for the duration of the study, unless otherwise noted. The colony room was maintained at 21–23◦C and kept under a reverse 12 h light/dark cycle (7:00 AM lights off). Additional environmental enrichment (i.e. acrylic hiding tubes, neoprene chew bones) was provided. All animals were treated in accordance with the National Institutes of Health Guide for the Care and Use of Laboratory Animals under a protocol approved by the Institutional Laboratory Animal Care and Use Committee at California State University, San Bernardino.

### Drug administration

Male and female Long-Evans rats were administered MPH 1 mg/kg (female = 10, male = 9) or 2 mg/kg (female = 9, male = 11) or vehicle control (female = 9, male = 8) for 10 days during adolescence on postnatal (PD) 30–39). A separate set of adult rats were also injected with control (female = 8, male = 8), MPH 1 mg/kg (female = 9, male = 9), or 2 mg/kg (female = 9, male = 9) on PD 80–89. MPH hydrochloride (Sigma-Aldrich, St. Louis, MO) was dissolved in 0.9% saline solution and administered intraperitoneally (i.p.) at a volume of 1 ml/kg twice a day (BID), at 9:00 after the lights turned off (7:00) and 8 h later in the evening (17:00) 2 h prior to lights on (19:00), to better mimic human exposure with more stable, prolonged administration. Control animals were injected with the 0.9% saline vehicle and treated in an identical manner as experimental groups.

### Activity monitoring

**Fig. 1:**
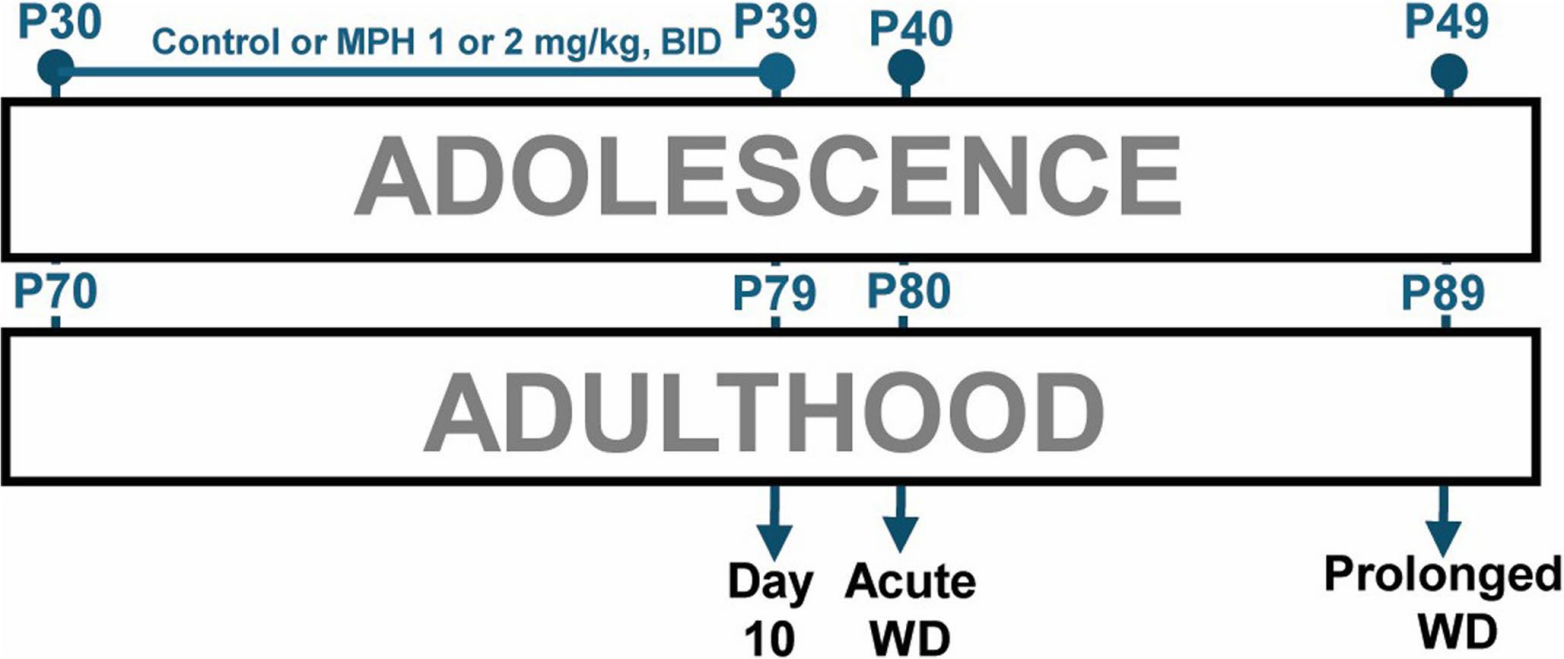
Overview of experimental timeline. Adolescent and adult rats were administered methylphenidate (MPH) two times a day (BID) with either 1, 2 mg/kg or vehicle control for 10 days (P30-39 or P70-79, respectively). Rats were assessed for minute-by-minute data over a 24 h period on the last day of administration (Day 10, beginning at dark onset on P39 or P79), the first 24 h without drug administration (acute WD, beginning at dark onset on P40 or P80), and 10 days after the last drug administration period (prolonged WD, beginning at dark onset on P49 or P89)

Rest-wake activity and circadian measures were collected using the Fitbit-like, non-invasive activity monitor called a FitBark^®^−2 (FitBark Inc. Kansas City, MO) which has been previously validated with further methodology provided in Ehlers et al. (2023). Briefly, rats were fitted with a rodent-sized harness or “jacket” made of blue spandex with the monitors placed in pockets on the dorsal side of the jacket via Velcro material (Lomir Biomedical Inc.). Rats were accustomed to the jackets for 12–16 h before each testing period. Activity monitors were then worn for 24 to 48 h to encompass an entire circadian (light/dark) cycle. Twenty-four hour recordings (beginning at 7:00) took place on the last day of MPH (adolescent PD 39–40 or adult PD 89–90), acute discontinuation (PD 40–41 or PD 90–91), and prolonged withdrawal (PD 49–50 or PD 99–100). A timeline of experimental design is provided in Fig. 1, outlining the 24 h activity days and MPH administration period for adolescent and adult rats.

Activity data from each animal was transferred via bluetooth to the FitBark^®^ app where raw data was available for download and further analysis. Data analysis methods use one-minute bins of FitBark^®^ activity data collected for the 24-hour periods which start at light offset (07:00 am). FitBark data was analyzed by two methods: (1) quantification of FitBark activity data and (2) cosinor analysis of circadian measures. Quantification of FitBark activity measures included overall mean activity at 3 h post-administration and 12 h period, number of episodes (also called bouts), maximum episode duration, mean episode duration, and fragmentation ratio (episode count/mean episode duration) in each 12 h light/dark period. Activity data was divided into active episodes defined as at least a 1 min bin with a cumulative activity score of over 0.2 as defined in Ehlers (2023) and 1 min bins of less than a 0.2 cumulative score were considered rest episodes. Cosinor analyses of combined 24 h light/dark periods included measures of MESOR (midline statistic of rhythm, or rhythm adjusted mean), amplitude, and acrophase (calculated in hours after light onset, 7:00) analyzed using a cosinor program (Molcan 2023). Acrophase is the time (hours) in which the peak value of activity occurred starting from 7:00 (0:00). Rats were individually housed for all recording periods.

For all analyses, 3-way ANOVAs were conducted for treatment (MPH 1 mg/kg, MPH 2 mg/kg, vs. control) x sex (female vs. male) x age (adolescent vs. adult) on each timepoint (Day 10, acute withdrawal, and prolonged withdrawal) for each behavioral measure. Significant effects were followed by 2-way ANOVAs and Tukey’s multiple comparisons post-hoc analyses as needed. Significance was set at *P* < 0.05.

## Results

### Mean activity during the dark period

**Fig. 2:**
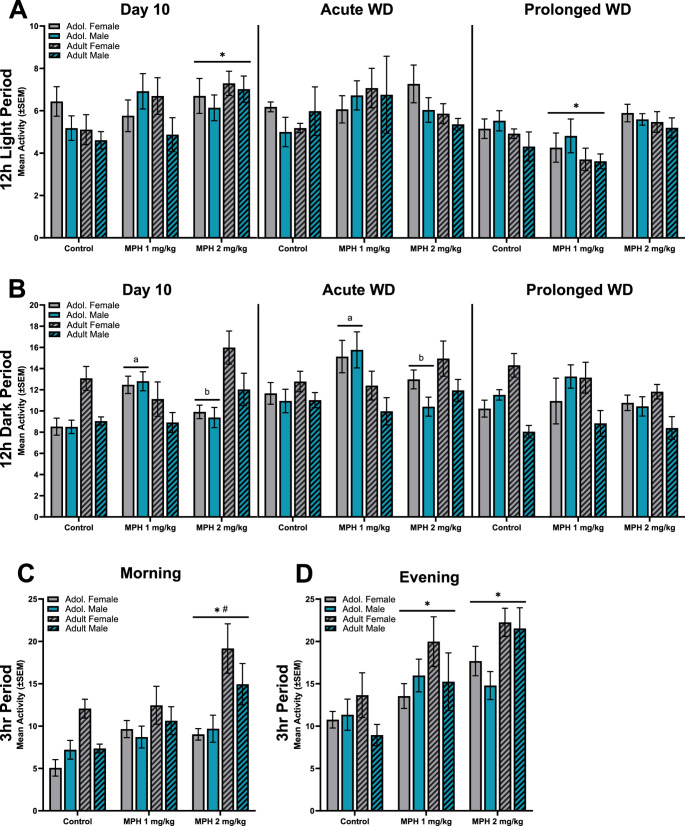
Overall mean activity of minute-by-minute cumulative data for the (**A**) 12 h dark period and (**B**) 12 h light period assessed on the last day of administration (Day 10, left panel), the first day of drug discontinuation (acute WD, middle panel), and after 10 days of discontinuation (prolonged WD, right panel). Male (blue bars) and female (grey bars), adolescent (plain bars) and adult (hatch bars) rats were treated with MPH (1, 2 mg/kg) or control and assessed at these three timepoints. Drug administration took place at 9:00 and 17:00 each day. Overall mean activity of minute-by-minute cumulative data was also taken on the last day of treatment during the (**C**) 3 h block after the morning administration (9:00–12:00) and (**D**) 3 h block after the evening administration (17:00–20:00). *vs. control, ^#^ vs. MPH 1 mg/kg, ^a^ vs. age-matched controls, ^b^ vs. age-matched MPH 1 mg/kg, *p* < 0.05

Overall mean activity of minute-by-minute cumulative data for the 12 h dark period (7:00–19:00) assessed across the three timepoints: the last day of administration (Day 10), the first day of drug discontinuation (acute WD), and after 10 days of discontinuation (prolonged WD) (Fig. 2A). A 3-way (treatment x age x sex) ANOVA of mean activity during the 12 h dark period on Day 10 (left panel), revealed a significant main effect of treatment [F (2, 97) = 4.004, *P* = 0.021], age [F (1, 97) = 5.462, *P* = 0.022], sex [F (1, 97) = 8.043, *P* = 0.006], treatment x age [F (2, 97) = 11.79, *P* < 0.001], and age x sex [F (1, 97) = 7.397, *P* = 0.008]. A follow-up 2-way (treatment x age) ANOVA revealed a significant main effect of treatment [F (2, 103) = 3.631, *P* = 0.030], age [F (1, 103) = 4.983, *P* = 0.028], and an interaction [F (2, 103) = 10.69, *P* < 0.001]. Post-hoc comparisons show that adolescent rats treated with MPH 1 mg/kg displayed significantly more activity during the dark period compared to controls (*P* < 0.001) and MPH 2 mg/kg (*P* = 0.021) treated adolescent rats. Additionally, adult rats treated with MPH 2 mg/kg displayed more activity during the dark period compared to controls (*P* = 0.026) and MPH 1 mg/kg treated adults (*P* = 0.002). An additional follow-up 2-way (sex x age) ANOVA revealed a significant main effect of sex [F (1, 105) = 6.806, *P* = 0.010], age [F (1, 105) = 4.186, *P* = 0.043], and an interaction [F (1, 105) = 5.713, *P* = 0.019]. Post-hoc comparisons show that adult female rats displayed significantly more activity during the dark period compared to adolescent females (*P* = 0.013) and adult male rats (*P* = 0.004). A 3-way (treatment x age x sex) ANOVA of activity during the 12 h dark period on acute WD (middle panel), revealed a significant main effect of sex [F (1, 87) = 4.925, *P* = 0.029] and treatment x age [F (2, 87) = 6.464, *P* = 0.002]. A follow-up 2-way (treatment x age) ANOVA revealed no main effects, but a significant interaction [F (2, 93) = 6.029, *P* = 0.004]. Tukey’s post-hoc comparisons show that adolescent rats treated with MPH 1 mg/kg displayed significantly more activity during the dark period compared to controls (*P* = 0.004) and MPH 2 mg/kg (*P* = 0.004) treated adolescent rats. A 3-way (treatment x age x sex) ANOVA of activity during the 12 h dark period on prolonged WD (right panel), revealed a significant main effect of sex [F (1, 95) = 6.967, *P* = 0.010] and age x sex [F (1, 95) = 18.120, *P* < 0.001]. A follow-up 2-way (age x sex) ANOVA revealed a main effect of age [F(1,103) = 7.250, *P* = 0.008] and an interaction [F (1,103) = 17.220, *P* < 0.001]. Tukey’s post-hoc comparisons show that adult male rats were significantly less active during the dark period compared to adult females (*P* < 0.001) and male adolescent rats (*P* = 0.005).

### Mean activity during the light period

Overall mean activity for the 12 h light period (19:00–7:00) was assessed across the same three timepoints (Fig. 2B). A 3-way (treatment x age x sex) ANOVA of activity during the 12 h light period on Day 10 (left panel), revealed only a significant main effect of treatment [F (2, 94) = 4.471, *P* = 0.014]. There were no other main effects or interactions between treatment, age, and sex. Tukey’s post-hoc comparisons show that irrespective of age or sex, rats treated with MPH 2 mg/kg displayed significantly more activity during the light period compared to controls (*P* = 0.001). A 3-way (treatment x age x sex) ANOVA of activity during the 12 h light period on acute WD (middle panel), revealed no significant differences. A 3-way (treatment x age x sex) ANOVA of activity during the 12 h light period on prolonged WD (right panel), revealed a significant main effect of treatment [F (2,93) = 8.609, *P* < 0.001] and age [F (1,93) = 5.332, *P* = 0.023]. There was no significant main effect of sex or any interactions between variables. Adolescent rats were overall more active during the light period compared to adult rats. Additionally, Tukey’s post-hoc comparisons showed that rats treated with MPH 1 mg/kg displayed significantly less activity during the light period compared to controls (*P* = 0.043) and MPH 2 mg/kg (*P* < 0.001) treated rats.

### Mean activity during the 3 h post administration

To determine whether MPH treatment increases initial locomotor activity after administration, data from 3 h post-administration during the morning (9:00–12:00) and evenings (17:00–20:00) was assessed for mean activity on Day 10. A 3-way (treatment x age x sex) ANOVA of the first 3 h of activity after morning administration on Day 10 (Fig. 2C), revealed a significant main effect of treatment [F(2,97) = 10.35, *P* < 0.001], age [F(1,97) = 23.090, *P* < 0.001], and age x sex interaction [F(1,97) = 4.926, *P* = 0.029]. Tukey’s post-hoc comparisons show that irrespective of age or sex, rats treated with MPH 2 mg/kg displayed significantly more activity after the morning injection on Day 10 compared to controls (*P* < 0.001) and MPH 1 mg/kg (*P* = 0.038) treated rats. A follow-up 2-way (age x sex) ANOVAs revealed a significant main effect of age [F (1, 105) = 18.23, *P* < 0.001] and an interaction [F(1,105) = 4.082, *P* = 0.046]. Post-hoc analysis found that adult female rats were more active after the morning administration compared to adolescent female rats (*P* < 0.001). A 3-way (treatment x age x sex) ANOVA of the first 3 h of activity after evening administration on Day 10 (Fig. 2D), revealed a significant main effect of treatment [F (2, 94) = 16.180, *P* < 0.001] and age [F (1, 94) = 6.240, *P* = 0.014]. Adult rats were also more active after the evening injection compared to adolescent rats. Tukey’s post-hoc comparisons showed that rats treated with MPH 1 mg/kg (*P* = 0.003) and MPH 2 mg/kg (*P* < 0.001) displayed significantly more activity after the evening injection on Day 10 compared to control rats.

### Active episodes during the dark period

**Fig. 3:**
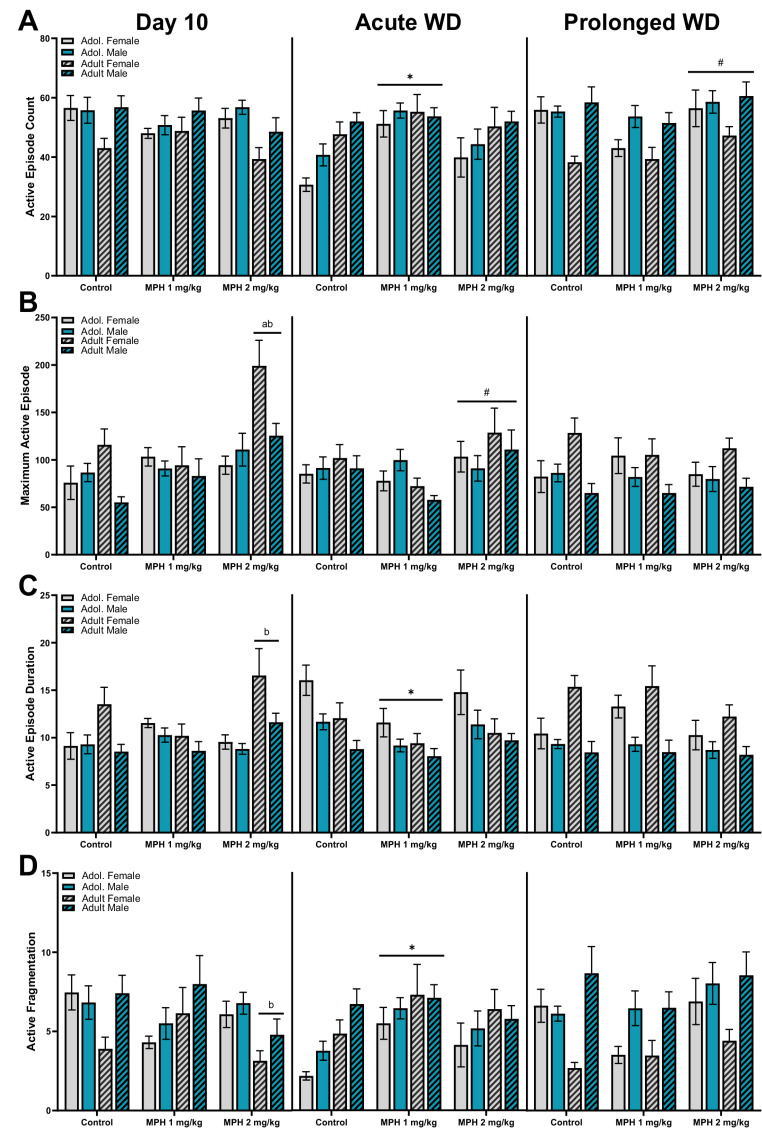
Active episodes were assessed during the 12 h dark period, when the animals spend more time awake. Male (blue bars) and female (grey bars), adolescent (plain bars) and adult (hatch bars) rats were treated with MPH (1, 2 mg/kg) or control were assessed on the last day of administration (Day 10, left panel), the first day of drug discontinuation (acute WD, middle panel), and after 10 days of discontinuation (prolonged WD, right panel). For the active episodes, the variables assessed across the three timepoints included the (**A**) mean number or count of active episodes, (**B**) maximum duration of active episodes, (**C**) mean active episode duration, and (**D**) the active fragmentation ratio. * vs. control, ^#^ vs. MPH 1 mg/kg, ^a^ vs. age-matched controls, ^b^ vs. age-matched MPH 1 mg/kg group. *p* < 0.05

As nocturnal animals, rats tend to be more active when the lights are off (dark period). As shown in Fig. 3, active episodes were investigated during the 12 h dark period (7:00–19:00) on three separate days: Day 10 of administration (left panels), first day of withdrawal from treatment (Acute WD, middle panels), and after 10 days of withdrawal from treatment (Prolonged WD, right panels). All activity data from the dark period is also included in Table 1.

**Table 1:**
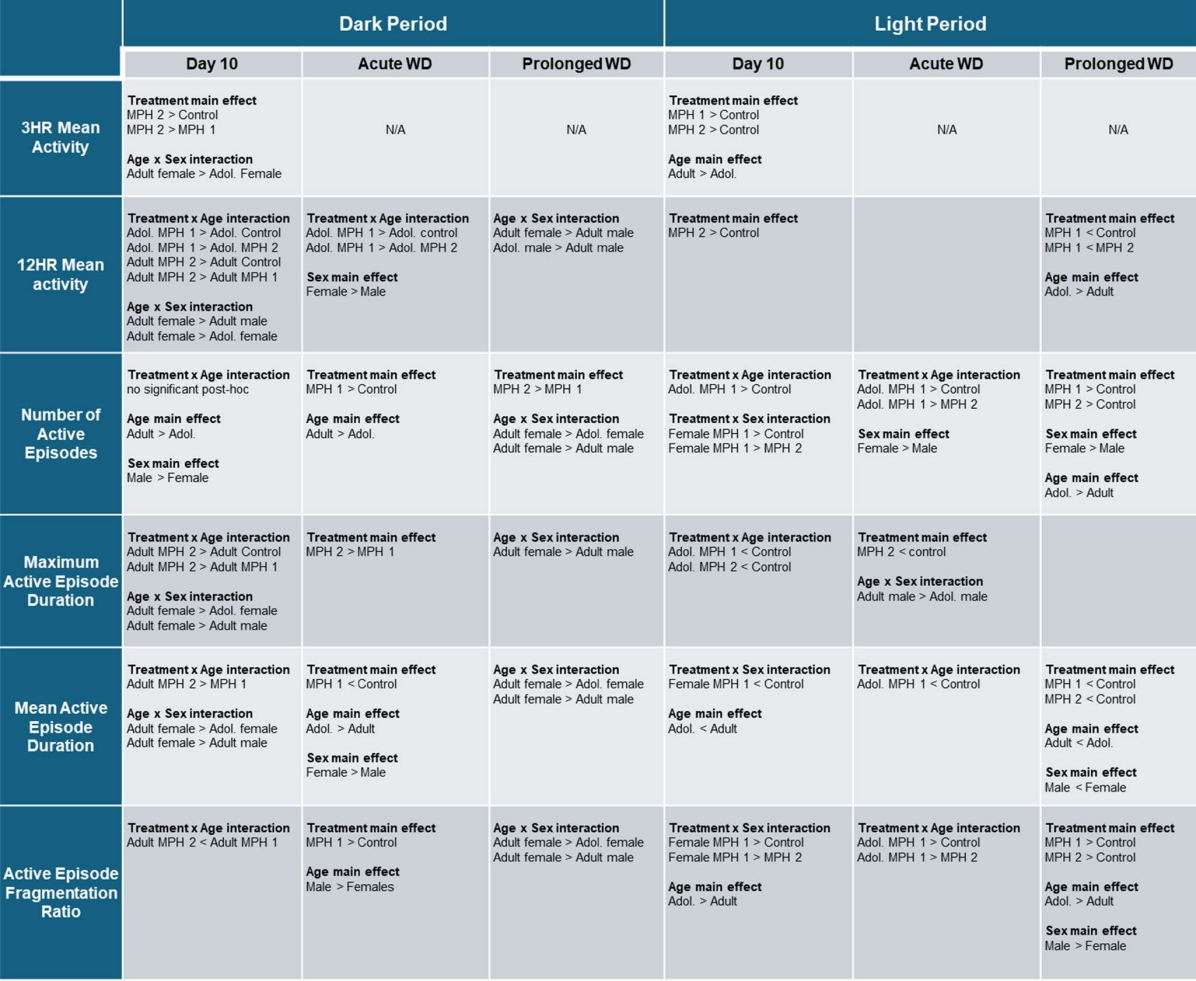
Summary of results from data collected during the dark and light periods

Significant main effects and interactions from each 3-way ANOVA are presented with significant post-hoc results for data collected during the 12hr dark and light periods. Methylphenidate (MPH, 1 mg/kg or 2 mg/kg) or control (saline vehicle) was administered for 10 days on PD 30-39 (adol., adolescent) or PD80-89 (adult) and assessed on day 10, acute withdrawal (WD), and prolonged WD

#### Number of active episodes

Separate three-way (age x sex x treatment) ANOVAs were conducted on the mean number (or count) of active episodes during the dark period for each of the three timepoints (Fig. 3A). For the number of active episodes that took place during the dark period on Day 10 (left panel), a 3-way (treatment x age x sex) ANOVA revealed a significant main effect of age [F (1, 97) = 4.995, *P* = 0.028], sex [F (1, 97) = 7.566, *P* = 0.007], and age x treatment interaction [F (2, 97) = 3.575, *P* = 0.032]. There was no significant main effect of treatment or other interactions for the number of active episodes. Male rats displayed more active episodes compared to female rats. A follow-up 2-way (age x treatment) ANOVAs revealed a significant main effect of age [F (1, 103) = 4.663, *P* = 0.033] but not treatment. There was a significant interaction [F (2, 103) = 3.411, *P* = 0.037], but no significant post-hoc analyses. For the number of active episodes that took place during the dark period on acute WD (middle panel), a 3-way (treatment x age x sex) ANOVA revealed a significant main effect of treatment [F (2, 93) = 5.825, *P* = 0.004] and age [F (1, 93) = 9.060, *P* = 0.003]. There was no significant main effect of sex or any interactions between the three variables. A Tukey’s post-hoc analysis revealed that irrespective of sex or age, MPH 1 mg/kg treated rats displayed more active episode counts than controls (*P* = 0.003). Additionally, adult rats overall displayed more active episode counts compared to adolescent rats. For the number of active episodes that took place during the dark period on prolonged WD (right panel), a 3-way (treatment x age x sex) ANOVA revealed a significant main effect of treatment [F (2, 96) = 4.643, *P* = 0.012], sex [F (1, 96) = 16.71 *P* < 0.001], and age x sex interaction [F (1, 96) = 5.573, *P* = 0.020]. There was no significant main effect of age or any other interactions for the number of active episodes. A Tukey’s post-hoc analysis revealed that irrespective of sex or age, MPH 2 mg/kg treated rats displayed more active episode counts than MPH 1 mg/kg treated rats (*P* = 0.008). A follow-up 2-way (age x sex) ANOVA revealed a significant main effect of age [F (1, 104) = 4.338, *P* = 0.040], sex [F (1, 104) = 15.44, *P* = 0.0002], and an interaction [F (1, 104) = 6.055, *P* = 0.0155]. Tukey post-hoc analysis revealed that adult female rats display significantly more active episode counts compared to adolescent female rats (*P* = 0.011) and adult male rats (*P* < 0.001).

#### Maximum active episode duration

Separate three-way (age x sex x treatment) ANOVAs were conducted on the mean maximum active episode duration during the dark period for each of the three timepoints (Fig. 3B). For the maximum active episode duration that took place during the dark period on Day 10 (left panel), a 3-way (treatment x age x sex) ANOVA revealed a significant main effect of treatment [F (2, 97) = 11.32, *P* < 0.0001], age [F (1, 97) = 4.306, *P* = 0.0406], sex [F (1, 97) = 5.998, *P* = 0.0161], and an interaction between age x treatment [F (2, 97) = 5.519, *P* = 0.0054] and age x sex [F (1, 97) = 9.049, *P* = 0.0033]. A follow-up 2-way (age x treatment) ANOVAs revealed a significant main effect of treatment [F (2, 103) = 9.906, *P* = 0.0001], but not age, and a significant interaction [F (2, 103) = 4.874, *P* = 0.0095] with adult rats treated with MPH 2 mg/kg displaying longer maximum active episodes compared to controls (*P* < 0.001) and MPH 1 mg/kg (*P* < 0.001) treated adults. An additional follow-up 2-way (age x sex) ANOVAs revealed a significant main effect of sex [F (1, 105) = 4.721, *P* = 0.032] and an interaction [F (1, 105) = 6.871, *P* = 0.010]. There was no main effect of age. Tukey post-hoc analysis revealed that adult female rats display significantly longer maximum active episodes compared to adolescent female rats (*P* = 0.013) and adult male rats (*P* = 0.006). For the maximum active episode duration that took place during the dark period on acute WD (middle panel), a 3-way (treatment x age x sex) ANOVA revealed a significant main effect of treatment [F (2, 94) = 4.578, *P* = 0.013] but no other significant main effects or interactions between age, sex, and treatment. Post-hoc comparisons revealed MPH 2 mg/kg treated rats display significantly longer maximum active episode durations on acute withdrawal compared to MPH 1 mg/kg treated rats. For the maximum active episode duration that took place during the dark period on prolonged WD (right panel), a 3-way (treatment x age x sex) ANOVA revealed a significant main effect of sex [F (1, 95) = 13.28, *P* = 0.0004] and an interaction between age x sex [F (1, 95) = 6.842, *P* = 0.0104] but no significant main effect of treatment, age, or an interaction between the three variables. A follow-up 2-way (age x sex) ANOVAs revealed a significant main effect of sex [F (1, 103) = 13.32, *P* < 0.001], and an interaction [F (1, 103) = 7.499, *P* = 0.007]. Post-hoc analysis revealed that adult female rats display significantly longer maximum active episodes compared to adult male rats (*P* < 0.001).

#### Mean active episode duration

Separate three-way (age x sex x treatment) ANOVAs were conducted on the mean active episode duration during the dark period for each of the three timepoints (Fig. 3C). For the mean active episode duration that took place during the dark period on Day 10, a 3-way (treatment x age x sex) ANOVA revealed no significant main effect of treatment but a significant main effect of age [F (1, 97) = 5.548, *P* = 0.0205], sex [F (1, 97) = 9.098, *P* = 0.0033], treatment x age [F (2, 97) = 6.314, *P* = 0.0026], and age x sex [F (1, 97) = 4.760, *P* = 0.0315]. A follow-up 2-way (treatment x age) ANOVA revealed a significant main effect of sex [F (1, 103) = 4.976, *P* = 0.028], and an interaction [F (2, 103) = 5.800, *P* = 0.004]. There was no significant main effect of treatment. Tukey post-hoc analysis revealed that adult rats treated with MPH 2 mg/kg displayed significantly longer mean active episode durations compared to adult rats treated with MPH 1 mg/kg (*P* = 0.002). Another follow-up 2-way (age x sex) ANOVA revealed a significant main effect of age [F (1, 105) = 190.1, *P* < 0.001], sex [F (1, 105) = 129.2, *P* < 0.001], and an interaction [F (1, 105) = 118.3, *P* < 0.001]. Post-hoc analysis revealed that adult female rats display significantly longer mean active episodes compared to adolescent female rats (*P* < 0.001) and adult male rats (*P* < 0.001). For the mean active episode duration that took place during the dark period on acute WD (middle panel), a 3-way (treatment x age x sex) ANOVA revealed a significant main effect of treatment [F (2, 93) = 3.758, *P* = 0.0270], age [F (1, 93) = 11.12, *P* = 0.001], and sex [F (1, 93) = 10.35, *P* = 0.0018]. There were no significant interactions across treatment, age, and sex. Post-hoc comparisons revealed that irrespective of sex or age, MPH 1 mg/kg treated rats displayed shorter mean active episode durations compared to controls during acute withdrawal (*P* = 0.028). Additionally, female rats had longer active episode durations compared to males, and adolescent rats were active for longer episodes compared to adults. For the mean active episode duration that took place during the dark period on prolonged WD (right panel), a 3-way (treatment x age x sex) ANOVA revealed a significant main effect of sex [F (1, 97) = 30.78, *P* < 0.0001] and age x sex interaction [F (1, 97) = 6.437, *P* = 0.0128]. There was no significant main effect of treatment, age or any other interactions. A follow-up 2-way (age x sex) ANOVA revealed a significant main effect of sex [F (1, 105) = 29.96, *P* < 0.001] and an interaction [F (1, 105) = 6.923, *P* = 0.010]. There was no main effect of age. Tukey post-hoc analysis revealed that adult female rats display significantly longer mean active episodes compared to adolescent female rats (*P* = 0.021) and adult male rats (*P* < 0.001).

#### Active episode fragmentation ratio

Separate three-way (age x sex x treatment) ANOVAs were conducted on the mean active fragmentation ratio during the dark period for each of the three timepoints (Fig. 2D). For the mean active fragmentation that took place during the dark period on Day 10 (left panel), a 3-way (treatment x age x sex) ANOVA revealed a significant main effect of sex [F (1, 97) = 5.042, *P* = 0.027], and treatment x age [F (2, 97) = 5.298, *P* = 0.007]. There was no significant main effect of treatment, age or any other interactions between the three variables. Males display more fragmented activity compared to females. A follow-up 2-way (treatment x age) ANOVA revealed no significant main effects (age or treatment) but there was a significant interaction between the two [F (2, 103) = 5.220, *P* = 0.0069]. Post-hoc comparisons show that adult rats treated with MPH 2 mg/kg displayed significantly less fragmented activity compared to MPH 1 mg/kg treated adults on Day 10 (*P* = 0.014). For the mean active fragmentation ratio that took place during the dark period on acute WD (middle panel), a 3-way (treatment x age x sex) ANOVA revealed a significant main effect of treatment [F (2, 93) = 4.213, *P* = 0.018] and age [F (1, 93) = 8.674, *P* = 0.004]. There was no significant main effect of sex or any interaction between the three variables. Follow-up comparisons demonstrate that irrespective of age or sex, rats treated with MPH 1 mg/kg displayed more fragmented activity during acute withdrawal compared to controls (*P* = 0.013). Additionally, male rats overall displayed significantly more active fragmentation compared to females. For the mean active fragmentation ratio that took place during the dark period on prolonged WD (right panel), a 3-way (treatment x age x sex) ANOVA revealed a significant main effect of sex [F (1, 96) = 17.61, *P* < 0.001] and age x sex [F (1, 96) = 5.739, *P* = 0.0185]. There was no significant main effect of treatment, age, or any interaction between the three variables. A follow-up 2-way (age x sex) ANOVA revealed a significant main effect of age [F (1, 105) = 10.85, *P* = 0.001], sex [F (1, 105) = 44.97, *P* < 0.001], and an interaction [F (1, 105) = 23.16, *P* < 0.001]. Post-hoc analysis revealed that adult female rats display significantly more fragmented active episodes compared to adolescent female rats (*P* < 0.001) and adult male rats (*P* < 0.001).

### Rest episodes during the light period

**Fig. 4:**
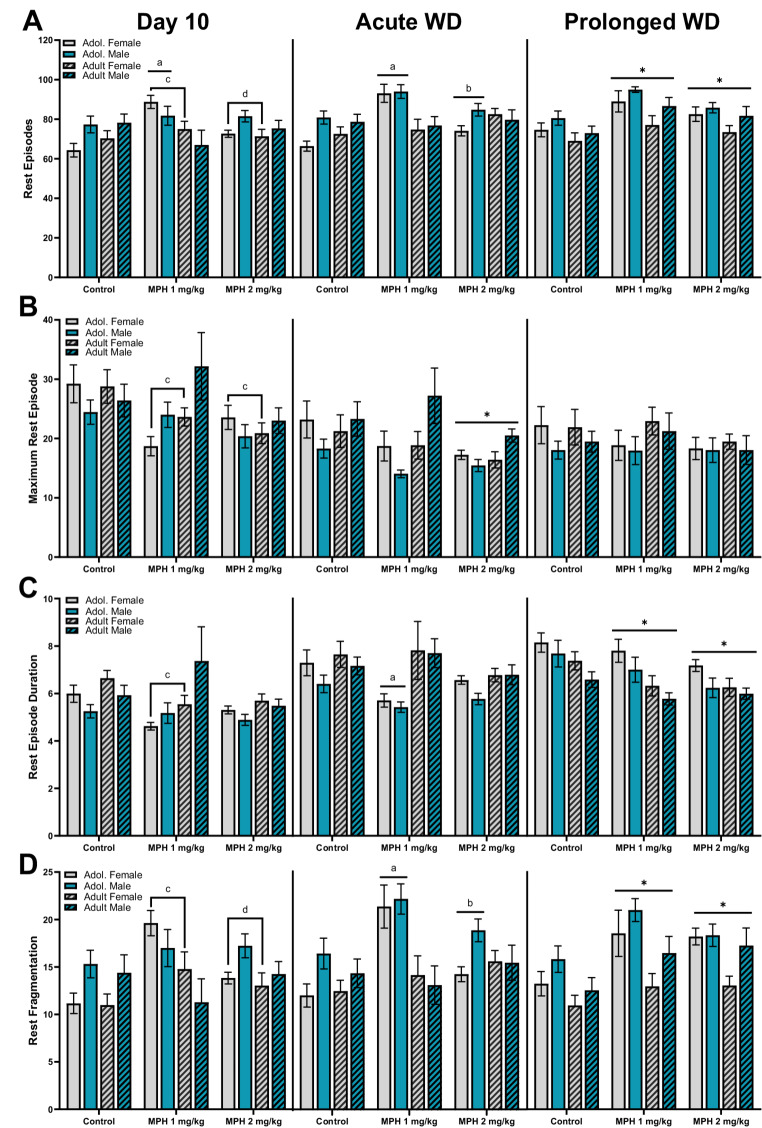
Rest episodes were assessed during the 12 h light period, when the animals spend more time asleep. Male (blue bars) and female (grey bars), adolescent (plain bars) and adult (hatch bars) rats were treated with MPH (1, 2 mg/kg) or control were assessed on the last day of administration (Day 10, left panel), the first day of drug discontinuation (acute WD, middle panel), and after 10 days of discontinuation (prolonged WD, right panel). For the rest episodes, the variables assessed across the three timepoints included the (**A**) mean number or count of rest episodes, (**B**) maximum duration of rest episodes, (**C**) mean rest episode duration, and (**D**) the rest fragmentation ratio. *vs. control, ^a^ vs. age-matched controls, ^b^ vs. age-matched MPH 1 mg/kg, ^c^ vs. sex-matched controls, ^d^ vs. sex-matched MPH 1 mg/kg. *p* < 0.05

As nocturnal animals, rats tend to be less active when the lights are on (12 h light period). Rest episodes are when the animal is moving minimally, as defined in the methods. As shown in Fig. 4, rest episodes were investigated during the 12 h light period (19:00–7:00) on three separate days; Day 10 of administration (left panels), first day of withdrawal from treatment (Acute WD, middle panels), and after 10 days of withdrawal from treatment (Prolonged WD, right panels). All activity data from the light period is also included in Table 1.

#### Number of rest episodes

Separate three-way (age x sex x treatment) ANOVAs were conducted on the mean number (or count) of active episodes during the light period for each of the three timepoints (Fig. 4A). For the number of rest episodes that took place during the light period on Day 10 (left panel), a 3-way (treatment x age x sex) ANOVA revealed a significant main effect of age [F (1, 93) = 4.379, *P* = 0.039], treatment x age [F (2, 93) = 4.824, *P* = 0.010], and treatment x sex [F (2, 93) = 5.305, *P* = 0.007]. There was no significant main effect of sex, treatment or any other interactions for the number of rest episodes. A follow-up 2-way (age x treatment) ANOVAs revealed a significant interaction [F (2, 99) = 4.422, *P* = 0.015] with adolescent rats treated with MPH 1 mg/kg displaying increased rest episodes count compared to age-matched controls (*P* = 0.001). There was no main effect of treatment or sex. Another follow-up 2-way (sex x treatment) ANOVAs revealed a significant interaction [F (2, 99) = 4.566, *P* = 0.013] with female rats treated with MPH 1 mg/kg displaying increased rest episodes count compared to sex-matched controls (*P* = 0.001) and MPH 2 mg/kg (*P* = 0.029). There was again no main effect of treatment or sex. For the number of rest episodes that took place during the light period on acute WD (middle panel), a 3-way (treatment x age x sex) ANOVA revealed a significant main effect of treatment [F (2, 91) = 6.529, *P* = 0.002], age [F (1, 91) = 4.365, *P* = 0.0395], sex [F (1, 91) = 5.435, *P* = 0.0219], and treatment x age [F (2, 91) = 8.113, *P* = 0.001]. Overall, female rats display more rest episodes than males. A follow-up 2-way (treatment x age) ANOVAs revealed a significant main effect of treatment [F (2, 97) = 6.100, *P* = 0.0032], age [F (1, 97) = 4.441, *P* = 0.0377], and interaction [F (2, 97) = 7.997, *P* = 0.001] with adolescent rats treated with MPH 1 mg/kg displaying increased rest episodes count compared to age-matched controls (*P* < 0.001) and MPH 2 mg/kg treated rats (*P* = 0.001). For the number of rest episodes that took place during the light period on prolonged WD (right panel), a 3-way (treatment x age x sex) ANOVA revealed a significant main effect of treatment [F (2, 95) = 10.74, *P* < 0.0001], age [F (1, 95) = 12.54, *P* = 0.0006], and sex [F (1, 95) = 7.889, *P* = 0.0060]. There were no significant interactions between treatment, age, and sex. Post-hoc comparisons show that irrespective of age and sex, MPH 1 mg/kg (*P* < 0.001) and MPH 2 mg/kg (*P* = 0.039) treated rats displayed significantly more rest episodes during the light period compared to controls. Additionally, female rats overall have more rest episodes than males, and adolescent rats overall have more episodes than adult rats.

#### Maximum rest episode duration

Separate three-way (age x sex x treatment) ANOVAs were conducted on the mean maximum rest episode duration during the light period for each of the three timepoints (Fig. 4B). For the maximum rest episode duration that took place during the light period on Day 10 (left panel), a 3-way (treatment x age x sex) ANOVA revealed a significant main effect of treatment [F (2, 93) = 4.578, *P* = 0.013] and treatment x age [F (2, 93) = 4.526, *P* = 0.013]. There was no significant main effect of age, sex or any other interaction between the three variables. A follow-up 2-way (age x treatment) ANOVAs revealed a significant main effect of treatment F (2, 99) = 4.553, *P* = 0.013], but no significant main effect of sex. There was a significant interaction [F (2, 99) = 3.989, *P* = 0.022] with adolescent rats treated with MPH 1 mg/kg (*P* = 0.004) and MPH 2 mg/kg (*P* = 0.020) displaying shorter maximum rest episodes compared to age-matched controls. For the maximum rest episode duration that took place during the light period on acute WD (middle panel), a 3-way (treatment x age x sex) ANOVA revealed a significant main effect of treatment [F (2, 88) = 3.755, *P* = 0.027], and age [F (1, 88) = 7.310, *P* = 0.008], and age x sex [F (1, 88) = 11.59, *P* = 0.0010]. Post-hoc comparisons show that irrespective of age or sex, MPH 2 mg/kg rats display significantly shorter rest episodes compared to controls (*P* = 0.021). A follow-up 2-way (age x sex) ANOVA revealed a significant main effect of age [F (1, 96) = 6.906, *P* = 0.010] and an interaction [F (1, 96) = 9.701, *P* = 0.002]. Post-hoc analysis revealed that adult male rats display significantly longer rest episodes compared to adolescent male rats (*P* = 0.001). For the maximum rest episode duration that took place during the light period on prolonged WD (right panel), a 3-way (treatment x age x sex) ANOVA revealed no significant main effects or interactions.

#### Mean rest episode duration

Separate three-way (age x sex x treatment) ANOVAs were conducted on the mean rest episode duration during the light period for each of the three timepoints (Fig. 4C). For the mean rest episode duration that took place during the light period on Day 10 (left panel), a 3-way (treatment x age x sex) ANOVA revealed no significant main effect of treatment or sex. However, there was a significant main effect of age [F (1, 93) = 13.79, *P* = 0.0003] and treatment x sex [F (2, 93) = 5.580, *P* = 0.005]. Adolescent rats displayed shorter rest durations compared to adult rats. A follow-up 2-way (treatment x sex) ANOVA revealed a significant interaction [F (2, 99) = 4.087, *P* = 0.020] with female rats treated with MPH 1 mg/kg display shorter mean rest episode durations compared to sex-matched controls (*P* = 0.011). There was no significant main effect of sex or treatment. For the mean rest episode duration that took place during the light period on acute WD (middle panel), a 3-way (treatment x age x sex) ANOVA revealed again no significant main effect of treatment or sex. However, there was a significant main effect of age [F (1, 90) = 15.49, *P* < 0.001] and treatment x age [F (2, 90) = 3.361, *P* = 0.039]. A follow-up 2-way (treatment x age) ANOVA revealed a significant main effect of age [F (1, 96) = 16.32, *P* < 0.001] and an interaction [F (2, 96) = 3.506, *P* = 0.034]. Tukey’s post-hoc analysis revealed that adolescent MPH 1 mg/kg treated rats display significantly shorter rest episode durations compared to age-matched controls (*P* = 0.023). For the mean rest episode duration that took place during the light period on prolonged WD (right panel), a 3-way (treatment x age x sex) ANOVA revealed a significant main effect of treatment [F (2, 95) = 7.268, *P* = 0.001], age [F (1, 95) = 17.64, *P* < 0.0001], and sex [F (1, 95) = 7.764, *P* = 0.006]. There were no significant interactions between the variables. Post-hoc comparisons show that irrespective of age and sex, MPH 1 mg/kg (*P* = 0.033) and MPH 2 mg/kg (*P* = 0.001) treated rats had shorter mean rest durations compared to controls. Additionally, adult rats overall had shorter rest durations compared to adolescents, and male rats overall had shorter rest durations in comparison to female rats.

#### Rest episode fragmentation ratio

Separate three-way (age x sex x treatment) ANOVAs were conducted on the mean rest fragmentation ratio during the light period for each of the three timepoints (Fig. 4D). For the mean rest fragmentation that took place during the light period on Day 10 (left panel), a 3-way (treatment x age x sex) ANOVA revealed a significant main effect of treatment [F (2, 92) = 3.269, *P* = 0.043], age [F (1, 92) = 8.720, *P* = 0.004] and treatment x sex [F (2, 92) = 5.441, *P* = 0.006]. There was no significant main effect of sex or any other interactions. Irrespective of sex or treatment, adolescent rats displayed more fragmented rest compared to adult rats. A follow-up 2-way (treatment x sex) ANOVA revealed a significant main effect of treatment [F (2, 98) = 4.268, *P* = 0.017] and an interaction [F (2, 98) = 4.892, *P* = 0.009]. There was no significant main effect of sex. Tukey post-hoc comparisons show that female rats treated with MPH 1 mg/kg displayed significantly more fragmented rest compared to controls (*P* < 0.001) and MPH 2 mg/kg (*P* = 0.021) treated female rats. For the mean rest fragmentation that took place during the light period on acute WD (middle panel), a 3-way (treatment x age x sex) ANOVA revealed a significant main effect of treatment [F (2, 90) = 5.756, *P* = 0.004], age [F (1, 90) = 12.92, *P* = 0.001], and treatment x age [F (2, 90) = 6.426, *P* = 0.003]. There is no significant main effect of sex or any other interactions. A follow-up 2-way (treatment x age) ANOVA revealed a significant main effect of treatment [F (2, 96) = 5.800, *P* = 0.004], age [F (1, 96) = 12.57, *P* = 0.001], and an interaction [F (2, 96) = 6.005, *P* = 0.004]. Post-hoc comparisons show that adolescent rats treated with MPH 1 mg/kg displayed significantly more fragmented rest compared to controls (*P* < 0.001) and MPH 2 mg/kg (*P* = 0.004) treated rats. For the mean rest fragmentation that took place during the light period on prolonged WD (right panel), a 3-way (treatment x age x sex) ANOVA revealed a significant main effect of treatment [F (2, 94) = 9.666, *P* < 0.001], age [F (1, 94) = 19.59, *P* < 0.001], and sex [F (1, 94) = 8.566, *P* = 0.004]. There were no significant interactions between these variables. Post-hoc comparisons show that irrespective of age and sex, MPH 1 mg/kg (*P* < 0.001) and MPH 2 mg/kg (*P* = 0.002) treated rats show significantly more fragmented rest compared to controls. Additionally, adult rats overall had lower rest fragmentation ratio compared to adolescents, and male rats overall had higher rest fragmentation in comparison to female rats.

### Circadian Measures

**Fig. 5:**
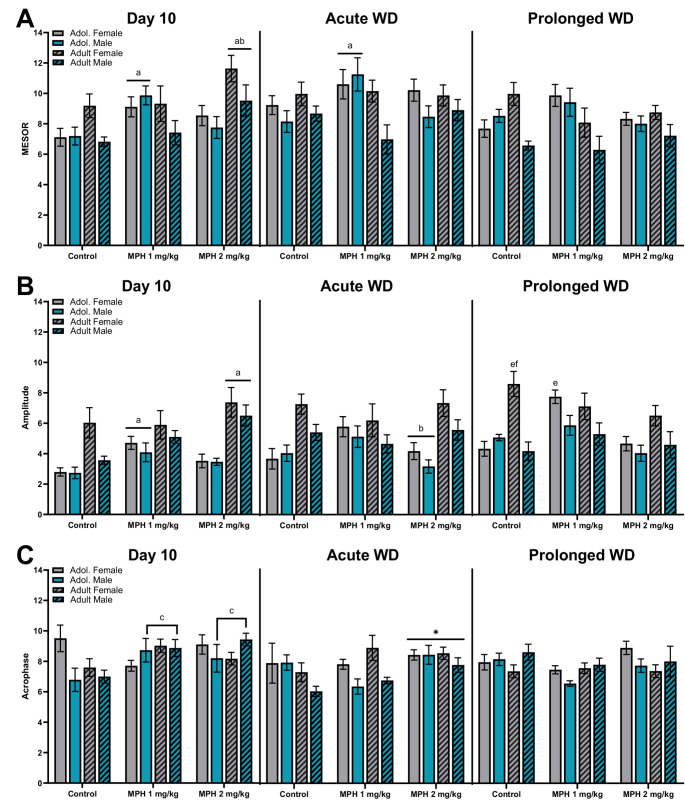
Circadian measures were assessed in male (blue bars) and female (grey bars), adolescent (plain bars) and adult (hatch bars) rats treated with MPH (1, 2 mg/kg) or control on the last day of administration (Day 10, left panel), during 24 h acute withdrawal (middle panel), and after 10 days of prolonged withdrawal (right panel). Circadian measures included (**A**) MESOR, (**B**) amplitude, and (**C**) acrophase. *vs. control, ^a^ vs. age-matched controls, ^b^ vs. age-matched MPH 1 mg/kg, ^c^ vs. sex-matched controls, *p* < 0.05

As depicted in Fig. 5, rats were assessed for changes in circadian measures after 10 days of MPH treatment (Day 10), during the first day of discontinuation from treatment (acute WD) and then 10 days of discontinuation (prolonged WD).

#### MESOR

Separate three-way (age x sex x treatment) ANOVAs were conducted on mesor (rhythm-adjusted mean) during 24 h period for each of the three timepoints (Fig. 5A). For the 24 h mesor taken on Day 10 (left panel), a 3-way (age x sex x treatment) ANOVA revealed a significant main effect of treatment [F (2, 91) = 6.039, *P* = 0.003], sex [F (1, 91) = 5.734, *P* = 0.019], treatment x age [F (2, 91) = 5.387, *P* = 0.006] and age x sex [F (1, 91) = 5.872, *P* = 0.017]. A follow-up 2-way (treatment x age) ANOVA revealed a significant main effect of treatment [F (2, 97) = 5.760, *P* = 0.004] and an interaction [F (2, 97) = 4.632, *P* = 0.012]. There was no significant main effect of age. Tukey’s post-hoc comparisons revealed adolescent MPH 1 mg/kg treated rats had significantly higher mesor compared to age-matched controls (*P* = 0.010) and adult MPH 2 mg/kg treated had significantly higher mesor compared to age-matched controls (*P* = 0.003) and MPH 1 mg/kg treated rats (*P* = 0.035). For 24 h mesor on acute WD (middle panel), a 3-way (age x sex x treatment) ANOVA revealed a significant main effect of sex [F (1, 92) = 7.660, *P* = 0.007] and treatment x age [F (2, 92) = 3.840, *P* = 0.025]. There was no significant main effect of treatment, age or any other interactions between variables. A follow-up 2-way (treatment x age) ANOVA revealed no main effect of treatment or age, but a significant interaction [F (2, 98) = 3.121, *P* = 0.049]. Tukey’s post-hoc comparison show that adolescent rats treated with MPH 1 mg/kg display higher mesor compared to age-matched controls (*P* = 0.020). For 24 h mesor on prolonged WD (right panel), a 3-way (age x sex x treatment) ANOVA revealed a significant main effect of age [F (1, 94) = 4.732, *P* = 0.032], sex [F (1, 94) = 8.522, *P* = 0.004], treatment x age [F (2, 94) = 4.409, *P* = 0.015], and age x sex [F (1, 94) = 8.896, *P* = 0.004]. There was no significant main effect of treatment or any other interactions between variables. A follow-up 2-way (treatment x age) ANOVA revealed no main effect of treatment or age. There was a significant interaction F (2, 100) = 3.388, *P* = 0.038], however, there were no significant post-hoc results. An additional follow-up 2-way (sex x age) ANOVA revealed a main effect of sex [F (1,102) = 7.449, *P* = 0.008] and an interaction [F (1,102) = 8.369, *P* = 0.005]. Tukey’s post-hoc analysis shows adult male rats had a significant lower mesor compared to adult female (*P* = 0.001) and adolescent male rats (*P* = 0.006).

#### Amplitude

Separate three-way (age x sex x treatment) ANOVAs were conducted on amplitude during 24 h period for each of the three timepoints (Fig. 4B). For the 24 h amplitude on Day 10 (left panel), a 3-way (treatment x age x sex) ANOVA revealed a significant main effect of treatment [F (2, 91) = 5.875, *P* = 0.004], age [F (1, 91) = 35.87, *P* < 0.001], sex [F (1, 91) = 4.944, *P* = 0.029], and treatment x age [F (2, 91) = 3.492, *P* = 0.035]. A follow-up 2-way (treatment x age) ANOVA revealed a significant main effect of treatment [F (2, 97) = 5.736, *P* = 0.0044], age [F (1, 97) = 34.95, *P* < 0.001] and an interaction [F (2, 97) = 3.262, *P* = 0.043]. Tukey’s post-hoc shows adolescent MPH 1 mg/kg rats have higher amplitude compared to adolescent controls (*P* = 0.030), and MPH 2 mg/kg treated adults have higher amplitude in comparison to adult controls (*P* = 0.003). For the 24 h amplitude on acute WD (middle panel), a 3-way (treatment x age x sex) ANOVA revealed a significant main effect of age [F (1, 92) = 19.35, *P* < 0.001], sex F (1, 92) = 7.388, *P* = 0.008], and treatment x age [F (2, 92) = 4.931, *P* = 0.009]. A follow-up 2-way (treatment x age) ANOVA revealed a significant main effect age [F (1, 98) = 19.92, *P* < 0.001] and an interaction [F (2, 98) = 4.633, *P* = 0.012]. Tukey’s post-hoc shows adolescent MPH 1 mg/kg rats have higher amplitude compared to adolescent MPH 2 mg/kg treated rats (*P* = 0.016). For the 24 h amplitude on prolonged WD (right panel), a 3-way (age x sex x treatment) ANOVA revealed a significant main effect of treatment [F (2, 94) = 5.794, *P* = 0.004], age [F (1, 94) = 4.168, *P* = 0.044], sex F (1, 94) = 19.86 *P* < 0.001], treatment x age [F (2, 94) = 3.297, *P* = 0.041], age x sex [F (1, 94) = 8.179, *P* = 0.005], and treatment x age x sex [F (2, 94) = 4.365, *P* = 0.015]. Tukey’s post-hoc analysis for treatment x age x sex interaction shows that adult female control rats had higher amplitude compared to adolescent female controls (*P* < 0.001) and adult male controls (*P* < 0.001). Additionally, adolescent female rats treated with MPH 1 mg/kg had significantly higher amplitude compared to adolescent female control rats (*P* = 0.020).

#### Acrophase

Separate three-way (age x sex x treatment) ANOVAs were conducted on acrophase during 24 h period for each of the three timepoints (Fig. 5C). For the 24 h acrophase on Day 10 (left panel), a 3-way (age x sex x treatment) ANOVA revealed no main effect and only a significant interaction between treatment x sex [F (2, 91) = 3.320, *P* = 0.041]. A follow-up 2-way (treatment x sex) ANOVA revealed no main effect of treatment or sex, but there was a significant interaction between the two [F (2, 97) = 3.114, *P* = 0.049]. Post-hoc analysis revealed that male rats treated with MPH 1 mg/kg (*P* = 0.014) and MPH 2 mg/kg (*P* = 0.008) had significantly delayed acrophase compared to sex-matched controls. For acrophase on acute WD (middle panel), a 3-way (age x sex x treatment) ANOVA revealed a significant main effect of treatment [F (2, 92) = 3.286, *P* = 0.042] and sex [F (1, 92) = 7.047, *P* = 0.009]. There was no significant main effect of age or any interactions. Female rats had significantly longer acrophase compared to males. Post-hoc analysis also shows that irrespective of sex or age, MPH 2 mg/kg treated rats have a delayed acrophase compared to controls (*P* = 0.049) after acute withdrawal. For acrophase on prolonged WD (right panel), a 3-way (age x sex x treatment) ANOVA revealed no significant main effects, with only one interaction between age x sex [F (1, 94) = 5.156, *P* = 0.025]. A follow-up 2-way (sex x age) ANOVA revealed no main effect of treatment or age. There was a significant interaction F (1, 102) = 5.202, *P* = 0.025], however, there were no significant post-hoc results.

## Discussion

The current study investigates the impact of the psychostimulant methylphenidate (MPH) on 24-hour sleep/wake activity patterns and circadian measures in adolescent and adult male and female rats. Results demonstrate that repeated MPH administration leads to dose- and age-specific increases in activity across the 12-hour light/dark cycle on the final day of treatment. This was accompanied by increased frequency and duration of active episodes during the dark period in MPH-treated adults, along with transient increases in fragmented activity during early withdrawal. MPH also reduced rest quality in female rats, evidenced by a decrease in the mean number of rest episodes and increased rest fragmentation during the light period. While some of these rest-related disruptions persisted immediately following discontinuation, more pronounced impairments in rest quality emerged 10 days after treatment cessation. These findings suggest that, although age- and sex-specific MPH treatment in a non-diseased model may increase activity during treatment, prolonged withdrawal may result in reduced sleep quality in both adolescents and adults exposed to psychostimulants.

Overall, MPH produced a dose-dependent increase in activity during 3-hour periods following both morning and evening injections, throughout the 12-hour dark phase, and notably during the 12-hour light period, when rats typically rest. Consistent with prior research, MPH (0.5–5.0 mg/kg) has been shown to stimulate locomotor activity (McNamara et al. 1993; Gaytan et al. 1996, 1997, 2000; Brandon et al. 2001; Dafny and Yang 2006; Lee et al. 2011; Yang et al. 2011), primarily via enhanced dopamine release in the nucleus accumbens (Segal and Kuczenski 1999; Medina et al. 2022). These dosages (0.5–3.5 mg/kg) correspond to clinically relevant plasma levels (Rush and Baker 2001). At higher doses (≥ 10 mg/kg), MPH can activate the dorsal striatum and elicit stereotyped behaviors (Kuczenski et al. 1997). Age- and sex-related differences in locomotor response to MPH have also been observed, with adults showing greater increases in activity following repeated MPH exposure compared to adolescents (Yang et al. 2011). Furthermore, chronic MPH administration results in greater sensitivity in females than males (Lee et al. 2009; Chelaru et al. 2012).

This study also identified dose-dependent increases in MESOR and amplitude, as well as a delayed acrophase on the final day of MPH treatment. The shift in acrophase in male rats persisted 24 h post-treatment but was no longer apparent 10 days after withdrawal. Consistent with previous literature, repeated MPH exposure has been shown to alter daily activity rhythms in a dose-dependent manner in both adult (Gaytan et al. 1997; Algahim et al. 2009, 2010; Lee et al. 2011; Trinh et al. 2013) and adolescent rats (Dafny and Yang 2006; Yang et al. 2006; Lee et al. 2009; Bergheim et al. 2012). MPH may influence circadian rhythms by delaying the onset of suprachiasmatic nucleus (SCN) electrical activity (Antle et al. 2012) and altering clock gene expression (Baird et al. 2013). Other psychostimulants have similarly been shown to shift striatal Per1 and Per2 gene expression, promoting a transition from nocturnal to diurnal activity patterns (Iijima et al. 2002). The current results indicate that while MPH can disrupt circadian measures, these effects are temporary and return to control levels within 10 days of withdrawal. However, circadian disruption, even for a short period of time, is linked to long-term health risks such as diabetes, cardiovascular disease, and hypertension (Karlsson et al. 2001; Bray and Young 2007; Lamont et al. 2007), highlighting the importance of understanding circadian rhythmicity as a potential biomarker of chronic psychostimulant exposure.

While prior studies have examined locomotor and circadian responses to MPH, this is the first to comprehensively characterize rest/wake episode patterns following psychostimulant exposure. Analyzing both active and rest episodes offers a more complete understanding of how stimulants impact 24-hour activity profiles. In this study, MPH-treated adult rats exhibited more sustained activity during the dark phase, including longer average and maximum active episode durations with less fragmented activity. These effects reversed during acute withdrawal, with shorter and more fragmented active episodes. During the light period, typically associated with rest, MPH treatment disrupted sleep-like behavior in females, reducing both maximum and average rest episode durations and increasing fragmentation. Acute withdrawal further decreased rest episode durations and increased rest fragmentation in adolescents. Although some changes were age- or sex-specific, prolonged withdrawal produced widespread reductions in rest episode number and average rest episode duration, indicative of sleep fragmentation. Sleep fragmentation, defined as frequent arousals that interrupt consolidated sleep, is known to impair memory, elevate neuroinflammation and stress, and disrupt metabolic and behavioral function in both humans and animal models (O’Brien and Gozal 2004; McCoy et al. 2007; Mavanji et al. 2012; Atrooz and Salim 2020; Dumitru et al. 2025). These findings underscore the potential translational relevance of sleep disturbances induced by MPH.

Patterns of activity seemed to differ between sexes, with females displaying generally more activity during the dark period, which included less frequent but longer active episodes, leading to less fragmented activity. Fewer sex differences were evident during the light period, with no differences in 12 h activity. However, females tended to have more frequent and longer rest episodes compared to males during this period. This aligns with previous research using FitBark activity monitors in Wistar rats (Ehlers et al. 2023), which found higher overall activity in female rats. Given the impact of estrous cycle on sleep-wake activity (for review see Swift et al. 2024), further investigations are necessary to determine whether fluctuations in sex hormones drive the MPH effects in female rats. However, changes in locomotor activity after administration of MPH (10, 20 mg/kg) during adolescence or young adulthood are not modulated by the phase of the estrous cycle in rats (Torres-Reverón and Dow-Edwards 2005).

While this study suggests MPH affects rest quality, it does not directly measure sleep architecture. Electroencephalogram (EEG) recordings provide a more precise tool for assessing sleep stages and brain activity. Although understudied, EEG-based research has demonstrated that acute MPH (10–30 mg/kg, p.o.) prolongs sleep latency and increases wake time in sleep-deprived rats (Ishida et al. 2010), with similar effects reported in mice (Antle et al. 2012). In Sprague Dawley rats, intravenous MPH (0.1–5.6 mg/kg) desynchronizes waking EEG, reducing power in alpha, beta, and gamma frequency bands (Zanettini et al. 2019). Both clinical and preclinical studies have shown that MPH alters sleep quality and quantity (Boonstra et al. 2007; Antle et al. 2012; Storebø et al. 2023). However, very few studies have investigated MPH’s effects on sleep/wake activity after treatment discontinuation. One clinical study of hospitalized individuals undergoing amphetamine withdrawal found that, following an initial phase of hypersomnia, sleep disturbances and variability in sleep duration persisted for over 20 days (Gossop et al. 1982). The mechanisms remain unclear, but neurotransmitter systems involved in attention and arousal also regulate sleep/wake states (Vetrivelan et al. 2010; Lazarus et al. 2013). One rodent study reported increased brain glucose metabolism in the lateral hypothalamus, a region critical to sleep/wake regulation, one week after a 13-week MPH regimen (4–10 mg/kg, p.o.) had been discontinued (Richer et al. 2022). Although attributed to feeding behavior (Martin et al. 2018), the role of the hypothalamus in sleep suggests possible long-term effects of psychostimulant exposure on arousal circuitry (Eban-Rothschild et al. 2018).

Given the increasing prevalence of early childhood psychostimulant use, there is growing interest in the long-term effects of MPH on the developing brain. Rodent studies suggest early MPH exposure produces long-lasting age-dependent neuroanatomical and behavioral changes (Urban et al. 2012; Urban and Gao 2015), including decreased sensitivity to natural and drug rewards (Bolaños et al. 2003; Amodeo et al. 2017), altered anxiety-like behavior (Crawford et al. 2003; Kelly et al. 2022), and changes in decision-making (Amodeo et al. 2017; Kelly et al. 2022). The present findings extend this literature, suggesting that MPH may also have lasting effects on sleep/wake patterns following discontinuation. Treatment discontinuation in ADHD individuals is highly prevalent, with reported rates ranging from 13 to 87%, with the highest attrition observed among young adults (for a review, see Brikell et al. 2024). Although treatment reinitiation is relatively common, overall pharmacological adherence and long-term use remain disproportionately low relative to ADHD prevalence. The current findings underscore the importance of understanding the long-term effects of methylphenidate (MPH) on sleep architecture and circadian regulation. Further research is needed to explore the complex relationship between early stimulant exposure, sleep, and neurophysiology, as well as the contribution of sleep/wake disturbances to long-term cognitive and reward-processing outcomes.

## Data Availability

The data that support the findings of this study are available from the corresponding author upon request.
